# A retrospective analysis of adrenal crisis in steroid-dependent patients: causes, frequency and outcomes

**DOI:** 10.1186/s12902-019-0459-z

**Published:** 2019-12-02

**Authors:** Katherine G. White

**Affiliations:** Institut für Politikwissenschaft und Soziologie, Julius-Maximilians-Universität Würzburg, Wittelsbacherplatz 1, 97074 Würzburg, Germany

**Keywords:** Adrenal insufficiency, Steroid medication, Glucocorticoid, Mineralcorticoid, Hydrocortisone, Fludrocortisone, Adrenal crisis, Intercurrent stress, Accident & Emergency, Ambulance

## Abstract

**Background:**

Adrenal patients have a lifelong dependency on steroid replacement therapy and are vulnerable to sudden death from undertreated adrenal crisis. Urgent treatment with parenteral steroids is needed, often with IV saline for volume repletion. Episodes of adrenal crisis are, for most patients, relatively infrequent and they may not be well prepared to respond. This study explores how patients recall previous episodes of adrenal crisis and their satisfaction with UK emergency medical treatment.

**Methods:**

We invited members of the main UK support groups representing steroid-dependent adrenal patients to complete an online questionnaire identifying the number, causes and location of previous adrenal crises (episodes needing injected steroids and/or IV fluids). Respondents were asked to rate the adequacy of their medical treatment in 2 successive questionnaires, conducted 2013 and 2017–18.

**Results:**

Vomiting was the major factor identified as a cause of adrenal crisis, indicated by 80% of respondents. The most common location, at 70%, was the home. Of the 30% away from home, 1 in 3 were overseas or travelling long-distance. Self-treatment played an increasing role in emergency response: in the 5 year interval between questionnaires an increasing number of patients self-injected. By the time of the 2017–18 survey self-injection was the most common method of initial treatment, with less than two-thirds travelling to hospital for follow-up medical treatment. This finding help to explain the higher rate of adrenal crisis identified in patient surveys than in hospital records. Satisfaction with medical care received stayed constant between the 2 surveys despite growing resourcing pressures across the NHS. Two-thirds were happy with the quality of the medical treatment they received for their most recent adrenal emergency; timeliness was the main factor influencing satisfaction.

**Conclusions:**

Around one-third of adrenal patients report sub-optimal treatment at emergency medical departments. Medical staff have a low probability of encountering adrenal crisis and may be unfamiliar with either the urgency of adrenal crisis or the specific treatment response it requires. Comprehensive protocols for emergency medical staff with detailed patient education and training are needed in how to respond to this infrequently encountered – but acutely life-threatening – scenario.

## Background

Emergency medical services across the UK have come under mounting pressure in recent years with year on year rises in attendance rates averaging 2% at major Accident & Emergency departments – an increase in excess of population growth [1]. Resource constraints imposed by central government funding ceilings have limited the ability of acute trusts to respond to rising public demands on Accident and Emergency services so that the proportion of patients waiting longer than 4 h rose from 4.3% in 2013–14 to 11.7% in 2017–18 [1].

Steroid-dependent patients are vulnerable to unanticipated episodes of life-threatening adrenal crisis, usually in association with vomiting that makes oral replacement therapy ineffective. Urgent treatment with high-dose injected steroids is needed, often with intravenous (IV) saline for volume repletion [2]. In the event of vomiting, early self-injection by the patient with intramuscular (IM) or subcutaneous (SC) hydrocortisone may prevent the need for more intensive hospital-based treatment [3]. Follow-up medical monitoring by emergency medical services is usually advised to ensure that the underlying causation has been resolved and IV fluids administered for volume repletion where required. Subcutaneous injection method for patient self-treatment had not been developed at the time of the 2013 questionnaire and, while now endorsed by French endocrine society as the method of choice [4–6] has yet to be recognised by the UK Society for Endocrinology; it is not widely known within the UK patient population that completed these questionnaires.

Steroid-dependence among Caucasian populations has an estimated prevalence of around 600 per million [7–9] and arises from two main causes: pituitary (secondary) insufficiency, including steroid-induced adrenal suppression, primary adrenal insufficiency, caused by autoimmune adrenal destruction or congenital adrenal hyperplasia, with other minor causes. Combined, all causes of steroid-dependence would give an estimated patient population approaching 40,000 across the UK.

Impaired pituitary function is the main cause of adrenal insufficiency, known as secondary adrenal insufficiency. The resulting loss of ACTH leads to reduced cortisol output by the adrenal glands and a daily dependence on replacement steroid therapy. This arises from a wide range of causes, most commonly pituitary adenoma and its treatment, but also head injury, haemorrhage or autoimmune hypophysitis. Diagnosed steroid suppression is included as secondary adrenal insufficiency, while recognising this is likely to underestimate the true incidence. Prevalence is estimated at around 400 cases per million.

Direct loss of adrenal gland function is a less common cause of adrenal insufficiency and is known as primary adrenal insufficiency. In most cases it is autoimmune or congenital but may also arise from surgical removal to treat uncontrolled Cushings’ syndrome or cancer, haemorrhage or infarction, or certain rare infections. Prevalence is estimated at between 100 and 220 per million for most European populations with higher frequency among Scandinavian populations [10].

Steroid-induced adrenal suppression may, over time, be reversible [11] while recovery from secondary adrenal insufficiency is more rare and from primary adrenal insufficiency almost unknown [12]. Patients have a lifelong dependency on replacement steroid therapy and remain vulnerable to sudden death from undertreated hypocortisolaemia from any cause as this can progress rapidly to acute adrenal crisis [13]. Coroner’s inquests into deaths from adrenal insufficiency and adrenal crisis – including nosocomial adrenal crisis among hospital inpatients – are a regular occurrence within the UK [14, 15] Previous studies have proposed adopting a broad and pragmatic definition of adrenal crisis to recognise that, left untreated, marked symptoms of adrenal insufficiency will usually progress to acute adrenal crisis and may result in sudden death from circulatory complications, cardiac arrest or organ failure [16–18].

Episodes of adrenal crisis are relatively infrequent for most adrenal patients. Previous studies have identified a frequency of hospital admission ranging from 3.3–10 per 100 patient years, with frequencies increasing over time [19–21]. Studies based on hospital records typically identify a lower adrenal crisis frequency than those which include medical treatment outside the acute setting or incidents of patient self-treatment without medical follow-up [4]. This study explores the range of circumstances in which patients describe experiences they perceive as adrenal crisis, the effectiveness of self-treatment and extent of patient reliance on various emergency medical resources, and the factors behind satisfaction with emergency medical services among adrenal patients.

## Methods

Members of the main UK support groups representing steroid-dependent patients were invited to complete an online questionnaire in 2013 and again in 2017–18, conducted through SurveyMonkey. Respondents were asked to identify the frequency, causes and location of their adrenal emergency experiences, which were defined as any episodes needing injected steroids and/or intravenous fluids. Respondents were also asked to describe the nature and timeliness of their medical treatment, as well as to provide demographic information that explored predisposing factors for adrenal crisis.

Preliminary focus groups and a small pilot survey identified definitional ambiguity as to what constituted adrenal crisis. Some respondents were reluctant to self-identify in this category even for incidents where they were severely hypotensive and semi-conscious, requiring ambulance transportation and hospital treatment with parenteral steroids. Conversely, others who had self-treated with oral steroids, in some cases surviving an extended period of vomiting without either parenteral steroid or medical attention, chose to identify this as an experience of adrenal crisis, presumably because their symptoms had been profoundly debilitating. The questionnaire invitation and many of the question wordings therefore used the term “adrenal emergency” to encourage a broad participation rate, while defining adrenal crisis or emergency as any incident that had required treatment with injected steroids.

Both questionnaires were comparatively lengthy for an online format and the licensed software utilised did not allow respondents to save their answers, log out and return later. To reduce the drop-out rate, skip logic was incorporated so that respondents declaring they had no prior experience of adrenal crisis were directed to later sections, which included medication regime and demographics, without viewing any of the questions about circumstances of adrenal crisis. The questionnaire was also structured not to require an answer to each question before progressing to the next one, so that respondents were able to skip over any question they perceived as not relevant, difficult or potentially distressing, while still completing the demographic section at the back of the questionnaire.

Members of the Addison’s Disease Self-Help Group (ADSHG), Association for Multiple Endocrine Neoplasia (AMEND), Living with CAH and the Pituitary Foundation gave responses in 2013. The questionnaire remained open from April 2013 to year end, with the largest proportion of the total 1054 responses submitted during late spring. The following 2017–18 questionnaire received 746 responses, some from endocrine patients not subscribing to any of the national charities although with a majority from ADSHG. Respondent demographics are summarised in Table 1. The two questionnaires adopted largely consistent wording but were not identical; the 2017–18 questionnaire asked respondents to identify their country of residence and which support group they belonged to; it asked additional questions about length of hospital admission, education in sick day rules and injection method.

**Table 1:** Respondent demographics for 2013 and 2017-18 online questionnaires

Cause of steroid-dependence	2013 *N* = 1053	2017–18 *N* = 746
Autoimmune adrenal failure (Addison’s)	51.5%	71.4%
Pituitary condition	31.9%	5.4%
CAH (congenital adrenal hyperplasia	5.9%	0.1%
Surgical removal of adrenals	3.5%	5.7%
Secondary adrenal suppression	2.9%	7%
TB, HIV or other infectious cause	1%	0.7%
Adrenal haemorrhage	0.1%	0.5%
ALD (adrenoleukodystrophy)	0.1%	0.2%
Don’t know	3.1%	4.2%
*All primary*	*62.1%*	*82.4%*
*All secondary*	*34.8%*	*12.4%*
Age, years, estimated mean	50 years	54 years
Age range	(0–10) – (90+) years	(0–10) – (90+) years
Years since diagnosis, estimated mean	11.5 years	12.4 years
Years since diagnosis: range	(Less than 1) – (50+)	(Less than 1) – (50+)
Female, %	73%	78%
Educated to tertiary level, %	60%	56%

The age profile of questionnaire respondents is more concentrated among 40–60 year olds than the UK population at large. This will partly reflect the fact that people are more likely to join a support group in the early years after diagnosis with a long-term medical condition, and the typical age of onset for the various conditions causing steroid-dependence [22]. A Swedish analysis of national medical registries identified an average age at diagnosis for autoimmune adrenal patients of 39.1 years [23].

Across the total sample, respondents reported an average of 11.5–12.2 years since diagnosis, with a small number (*N* = 5–9) self-reporting their diagnosis had taken place more than 50 years ago. The proportion of over 80s in the wider UK population is higher than in these surveys so that those with the greatest likelihood of needing emergency medical care may be under-represented in this analysis. The web-based technology used for this questionnaire may help to explain the gap as a smaller proportion of elderly householders have internet access than the wider UK population [24].

## Results

**Fig. 1:**
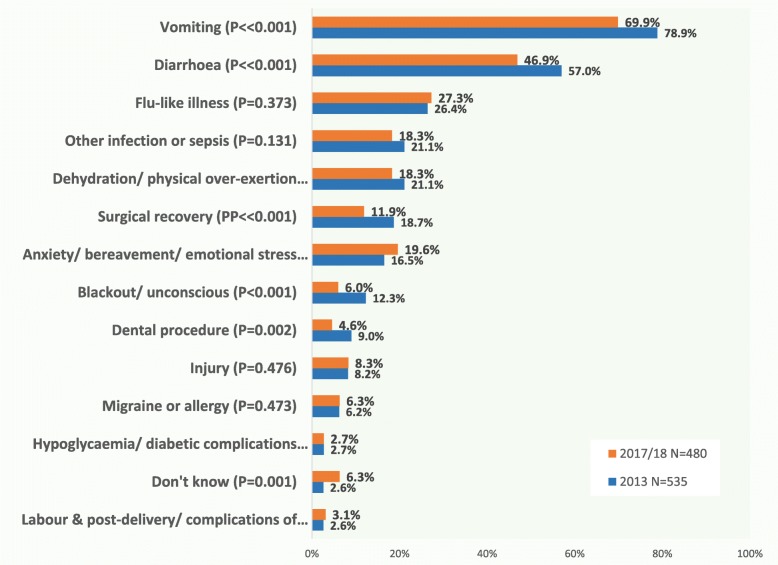
What factors have seemed to be a cause for the various adrenal emergences that you have experienced *(tick as many as apply)*

Vomiting was the predominant causative factor associated with episodes of adrenal crisis, identified by 70–80% of respondents as contributing to a previous episode (Fig. 1). Diarrhoea was the second most frequent factor, identified indicated by 46.9–57%. In combination, these suggest that infective gastro-enteritis is a major risk for adrenal insufficiency patients. Infection without vomiting, identified as flu-like illness (26.4–27.3%) and other infection or sepsis (18.3–21.1%) were the third and fourth most frequently reported causal factors. Under-treatment during surgical recovery was identified by a noticeable proportion (11.9% – 18.7) as having been a factor in a previous episode of adrenal crisis.

Respondents identified an average of 2.6–3.2 factors from the tick box list provided, so that in many incidents, trigger factors may be cumulative and vomiting may have been a symptom of hypocortisolaemia brought about by dehydration or physical over-exertion, emotional anxiety or distress, migraine, allergy or other causes rather than an infective gastroenteritis such as norovirus. One-third of those who attended hospital said they had required medical treatment for other conditions at the same time as their adrenal insufficiency, indicating a wide range of infections including pneumonia, urinary tract (UTI), diverticulitis and sepsis, various fractures and car crash injuries, complications of diabetes and asthma, as well as anti-emetics and anti-spasmodics for vomiting and diarrhoea. Injury was a comparatively minor causal factor, reported by just over 8% of respondents; write-in comments indicated that in some cases this was accompanied by emotional shock from involvement in a traffic accident.

**Fig. 2:**
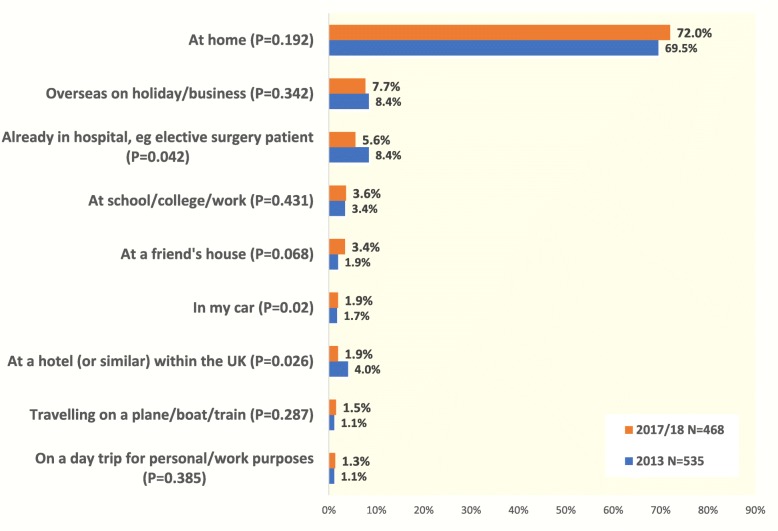
Where were you when you experienced your most recent adrenal emergency?

Around 30% of patients were away from home at the time of their most recent adrenal emergency, with almost 10% overseas or travelling long distance at the time (Fig. 2). This finding helps to explain the higher frequency of adrenal crisis identified in patient surveys than in hospital records, as these patients will not have been treated at their regular hospital. It underlines the importance of ensuring that all adrenal patients are issued with an emergency injection kit for personal use, with training for family members as well as patients in how to use it.

**Fig. 3:**
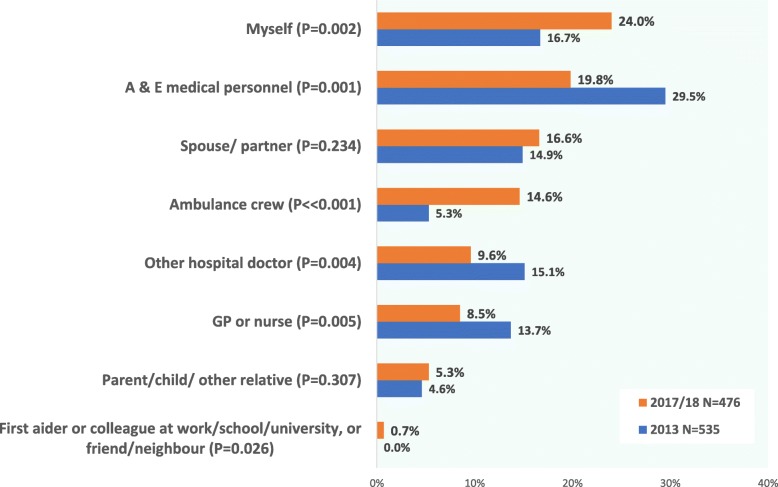
For your most recent adrenal emergency, who gave you a hydrocortisone injection? (or IV hydrocortisone via a cannula)

The proportion of patients who either self-injected or were given injected hydrocortisone by a family member or ambulance crew increased significantly between the two surveys with a proportionate decrease among those whose initial treatment was administered by Accident and Emergency staff (Fig. 3). By the time of the 2017–18 questionnaire, a full 71% of adrenal patients reported receiving their initial treatment in a community setting – without treatment being delayed until they could be transported to hospital.

**Fig. 4:**
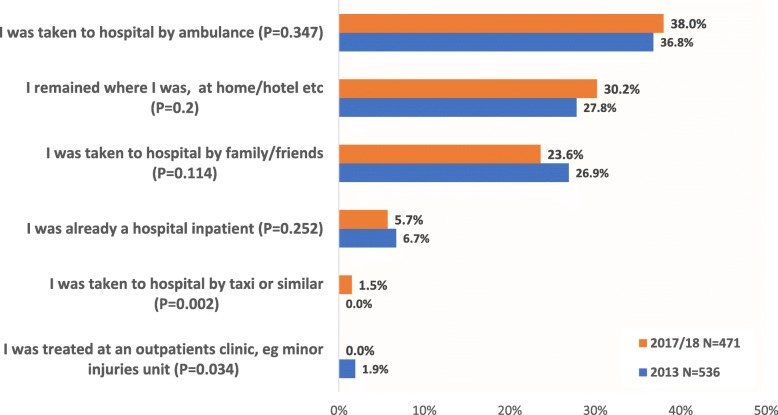
Did you travel to a hospital or medical centre for treatment or follow-up to your most recent adrenal emergency?

While many were initially stabilised in a community setting, in 2017–18, almost two-thirds of respondents said they were taken to hospital for their most recent adrenal crisis (Fig. 4). The proportion of patients transported to hospital by ambulance, at around 38%, is more than two-thirds higher than the average recorded for all A & E attendances in England, which was 22.2% for 2017–18 [25, 26]. Over one-quarter remained where they were; predominantly at home, but some in a hotel or elsewhere. Some of those who remained where they were reported being treated/assessed by an attending GP or practice nurse. Less than 2% were treated at a minor injuries unit or similar, while 5% said they were already a hospital inpatient and their adrenal crisis was iatrogenic or nosocomial – triggered by insufficient steroid medication during surgical recovery or post-labour.

The sizeable proportion whose emergency treatment for adrenal crisis took place outside the acute care setting helps to explain why patient surveys identify higher rates of adrenal crisis than identified from hospital records [27].

62% said that only their adrenal insufficiency had needed treating, while 38% said they needed treatment for other medical problems at the same time. Consistent with the trigger factors identified for adrenal crisis, pneumonia and other infections requiring IV antibiotic, vomiting requiring anti-emetics and anti-spasmodics were the most common medical problems descried in the write-in comments as needing treatment. Brittle asthma, poor control of diabetes, fractures, car accidents and other injuries, appendicitis or stroke were minor causes. 12% reported that there had been a delay while medical personnel located injection materials.

The 2017–18 survey asked respondents to identify the time to treat at Accident & Emergency, for their most recent adrenal emergency. 40% (*N* = 467) said they were treated in less than 30 min, and two-thirds in under an hour. Just under 20% said their treatment for adrenal crisis was delayed more than 2 h, of whom 7% said it took 4–8 h before they were treated and 2% more than 8 h. This means that around one-third of adrenal patients are receiving an inadequate medical response when they present at Accident & Emergency, compared to best practice advice from adrenal specialists [28].

**Fig. 5:**
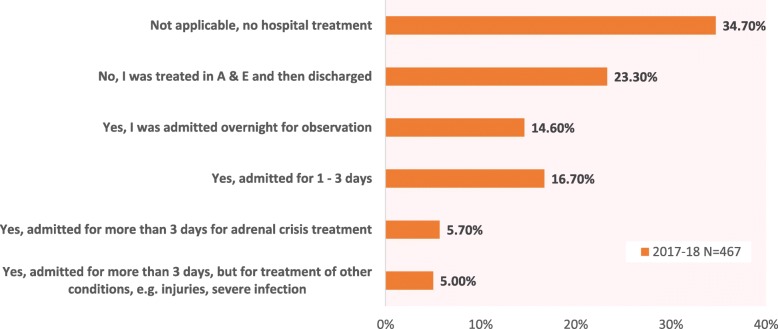
Did you need to be admitted for inpatient treatment following your most recent adrenal crisis?

In the 2017 questionnaire – but not in 2013 – respondents were asked to specify if they had received inpatient treatment for their most recent adrenal crisis, had been discharged from Accident and Emergency or not gone to hospital for treatment (Fig. 5). Of those who attended hospital, just under half were then admitted for inpatient treatment. Inpatient treatment for the majority lasted 1–3 days, with around 40% admitted overnight for observation, a treatment path which could be expected for a life-threatening condition. One-third said they had not attended hospital, and nearly a quarter had been treated at A & E then discharged. Thus, over 40% of recent adrenal crises reported by patients in this survey required hospital admission, most for 1–3 days.

Satisfaction levels were broadly in line with time to treat; just over two-thirds said they were very satisfied or satisfied with the quality of treatment for their most recent adrenal emergency. Despite growing resourcing pressures on the NHS across this five year interval, satisfaction rates were consistent in the two surveys: 66.7% in 2013 and 70.4% in 2017–18. The proportion expressing dissatisfaction reduced slightly, from 25.8% in 2013 to 20.5% in 2017–18.

Open comment responses to the 2013 prompt: *What were the best aspects of the medical treatment you received for your most recent adrenal emergency?* typically cited timeliness, familiarity with steroid-dependence and/or willingness to listen to the patient and their advocate as to what was needed. The one-quarter to one-fifth of respondents who felt their medical treatment had been inadequate were often unable to identify any positive aspects to the experience. Many expressed distress at poor recognition for their life-threatening condition. This open comment question was not repeated in the 2017–18 survey.

Both questionnaires asked respondents to indicate if they had needed emergency treatment for adrenal crisis within the past 12 months, or at any stage since their diagnosis, in an attempt to get a sense of the frequency of adrenal crisis. For these questions, adrenal insufficiency was simply defined as any event requiring injected steroids, either intravenous or intramuscular.

In 2013, 65% said they had not needed emergency treatment within the past 12 months, while 35% indicated 1 or more episodes of adrenal crisis within the past 12 months. Similar proportions recurred in 2017–18, with 63% indicating none and 37% identifying 1 or more episodes requiring emergency treatment within the past 12 months. In 2013, 63% indicated they had experienced 1 or more previous adrenal crises at some stage since their diagnosis, and in 2017–18 the proportion was 67%, with an average of 11.5–12.4 years since diagnosis. These proportions are considerably higher than previous studies. In a 2003 paper-based questionnaire, reported elsewhere [29], only 45% reported that had experienced a previous adrenal crisis (*N* = 864). These findings suggest self-selection, with a high proportion of patient charity members who had past experience of adrenal crisis being motivated to complete the online questionnaire and a smaller proportion of those who had enjoyed more stable health doing so. Therefore, this analysis does not attempt to interpret the frequency of adverse events from questionnaire responses.

## Discussion

Self-treatment played an increasing role in emergency response to adrenal crisis, with a noticeable rise in the number of respondents able to self-inject over the 5-year interval between the two surveys. By 2017–18, self-injection was the leading method of initial treatment, reported by 24% of respondents; more patients gave their own initial parenteral hydrocortisone than received this from Accident and Emergency medical staff (19.8%). In 2013, injection given by A & E staff had been the leading method at 29.5%. (Chart 3). Both surveys identify a striking increase on the proportion of patients able to self-inject compared to earlier patient surveys. Only 6% of replies to the 2003 paper questionnaire [30] said they had been able to self-inject for their most recent adrenal crisis. In 2017–18, four times as many – 24% – said they had self-injected (P < < 0.001). The proportion who were injected by family or friends was 8% in 2003; this almost tripled to 23% by 2017–18 (P < < 0.001). In 2003, 86% had received their initial parenteral hydrocortisone from medical staff. By 2017–18, that had scaled back to 52% who received their initial injection from a medical professional (P < < 0.001).

One factor likely to have contributed to this rise was the wider availability of online instruction regarding injection method. A number of UK hospitals published instructional leaflets or videos online over this period, including one made available as a free phone App [30]. Some endocrine clinics also developed group education programmes addressing emergency management and self-injection [31–33]. The UK adrenal charity involved in recruiting, ADSHG, developed several new patient education materials illustrating injection method over this period that are likely to have contributed to an improvement among respondents belonging to the same charity [34].

Over the same 5-year interval, the proportion reporting that they received their initial parenteral hydrocortisone from ambulance crew also increased markedly, rising nearly three-fold from 5 to 13.7% (*P* < < 0.001). Many UK ambulance trusts introduced new or extended educational programmes for their staff about steroid dependence and adrenal crisis during this interval and the national ambulance regulator launched an extended set of adrenal crisis guidelines in its 2017 supplementary guidelines [35]. The two main adrenal charities, Pituitary Foundation and ADSHG, both ran awareness programmes during these years encouraging their members to register their steroid-dependent condition with their local ambulance trust [36].

Time to treat identified in the 2017–18 survey is broadly consistent with the findings from two smaller German studies [26, 37]. However, 1 in 5 respondents experienced delays beyond the 2 h upper limit advised by the endocrinologists consulted in a German study [28]. Accident & Emergency departments thus need to be vigilant in ensuring triage and emergency nursing staff are informed about the time-critical needs of adrenal patients presenting with symptoms of adrenal crisis and prioritize their treatment. This should not be taken for granted, given the considerable resourcing pressures faced by most units. Recent initiatives at some teaching hospitals to introduce pop-up alerts on adrenal patient hospital records about the time-critical nature of emergency medical treatment and administration of glucocorticoid doses will undoubtedly assist in this respect [38]. A further promising safety initiative has been for direct ambulance registration of steroid-dependent patients by home address, with registration being arranged by their treating hospital [39].

**Fig. 6:**
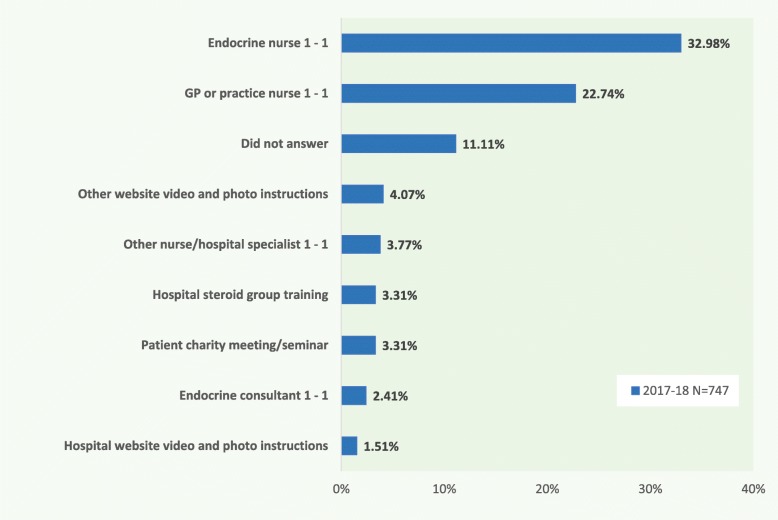
What training have you had in the use of your emergency injection kit? *(please tick as many as apply)*

The findings from this study about the proportion of adrenal patients who are able to self-treat with injected steroids, alongside those who required urgent medical attention through outpatients and/or hospitalisation, are not dissimilar to those found in a smaller scale, multi-centre prospective German study based on patient questionnaires [28]. Notably, 32.2% reported self-injection in the German study (*N* = 39), compared to 24% in the most recent British survey (*N* = 445). A further 15.3% reported that a family member gave the injection in the German study, compared to 23.3% in the British survey. Thus, in both questionnaires, around 47% of respondents reported that pre-hospital treatment for their most recent adrenal crisis was either self-administered or given by a family member (*P* = 0.505). Most of those completing the German questionnaire had received in-person training in a group education programme, where British respondents had a more diverse range of prior information or training opportunities. 62% of UK survey replies reported they had received individual training from either an endocrine consultant or nurse, other hospital specialist, GP or practice nurse, while 42% had viewed online video materials about injection method and 16% had taken part in a group training session (Fig. 6).

Three factors may have contributed to the high proportion of respondents to these questionnaires reporting a previous adrenal crisis. The first is that most previous analyses were drawn from hospital activity records for a single hospital catchment [13–15]. This study comprises self-reported episodes which include those treated in overseas medical units, as well as those who self-treated at home or received parenteral steroids at home from an on-call GP or ambulance crew and were assessed as stable enough not to require hospital follow-up. (Chart 3) Around 40% of the episodes in this survey would not have been treated at the patient’s usual hospital. Supporting this hypothesis are a German study of insurance records and an EU study of patient diary records [40, 41]. Both found a similar rate of adrenal crisis – around 10 per 100 patient years – which was higher than comparable German analyses of hospital records [14, 42], even if more than 3 times less than the proportion reported by respondents to this study.

A second factor is likely to be that patients with greater morbidity have stronger psychosocial needs and are more motivated to join a support group, producing a form of selection bias. Additionally, within the membership of the support groups that recruited for the 2013 and 2017–18 questionnaire, those with a recent experience of adrenal crisis would have been more motivated to log in and reply to the online questionnaire than those without a relevant personal experience to share.

Equally, patients belonging to a support group are perhaps likely to be more adept at adapting their oral glucocorticoid to self-treat during infections such as influenza, compared to patients without similar access to peer learning and charity educational resources. Support group members typically receive regular newsletters, email bulletins and social media updates as well as the opportunity to attend medical lectures and social discussion groups, so could be predicted to be better informed about – and more proficient at – self-management of dosage adjustments for intercurrent illness. This may have influenced the relatively low proportion in these 2 support group surveys who reported adrenal crisis from causes responsive to extra oral steroid medication, such as flu-like illness or other febrile infection. The German observational study described above [28] found that a higher proportion of adrenal crisis episodes were caused by febrile infections than by infections involving vomiting. Studies of patients in the Netherlands, Japan and Australia have all found higher rates of adrenal crisis requiring hospital treatment for influenza and other infections [43–46]. Thus, vomiting and diarrhoea could arguably be over-represented as a trigger factor among the UK support group members studied here, compared to wider patient samples.

Analysis of Australian hospital records (*N* = 824) also identified higher rates of adrenal crisis among the elderly [46]. Bacterial infection – predominantly pneumonia and UTI – was a major cause. Without attempting to control for years since diagnosis, the 2017–18 survey analysed here does not replicate the finding of increasing incidence or hospital admission with age. (The 2013 survey did not ask about admission rates or length of stay).

Among those aged 70 and over (*N* = 110), 65% reported one or more episodes of adrenal crisis in 2017–18, compared to a well-matched 67% of those under 70 years old (*N* = 592). Screening out those who were treated outside a hospital (in the community) for their most recent adrenal crisis, the proportion reporting one or more post-diagnosis adrenal crises in the 2017–18 survey rises to 100% among those aged 70 and over (*N* = 41), matched by a similar 100% among the under 70s. (*N* = 234). However, comparing self-reported trigger factors for all previous adrenal crises between the under 70s and those older, there are two main differences emerge from the 2017–18 survey. Those aged 70 and over were twice as likely to report either a peri-operative adrenal crisis triggered by insufficient glucocorticoid (*P* = 0.997; 21.7%; 10.3%) or adrenal crisis triggered by injury, presumably mostly falls (*P* = 0.975, 14.5%; 7.3%). This raises an uncomfortable question about ageism, as to whether elderly adrenal patients in hospital are less likely to be accepted as “expert patients’ by medical staff and their views on treatment needs more likely to be ignored. Resistance to patient-supplied treatment information is a factor that has been noted in other studies exploring inpatient treatment of steroid-dependence and iatrogenic hypoadrenalism [27]. It is possible that elderly adrenal patients are both less likely to enter hospital equipped with documentation of their treatment needs – possibly, especially where they are an emergency admission following a fall –and more likely to have their expressed views ignored.

The specific needs of elderly adrenal patients may be a fruitful area for further study, especially in light of a recent large-scale EU-AIR observational study. This identified adrenal crisis in combination with infection as a significant cause of death, particularly for older male patients with secondary adrenal insufficiency and diabetes mellitis plus hypertension as comorbidities, a cohort which they identified as experiencing more frequent adrenal crises [47]. The authors concluded that this high risk group, in particular, deserved further attention and care. As discussed above, this present study did not identify older patients (those over 70) as being at higher risk of adrenal crisis from causes other than surgical recovery or injury. However, there is unarguable merit in closer examination of the factors that might make certain adrenal patient cohorts more vulnerable to adrenal crisis, with its inevitable risk of sudden death from delayed or inadequate treatment.

One weakness of this study is that it contains no record of adrenal patients who died in adrenal crisis, data which is captured by hospital or insurance databases. Therefore, it is possible that certain causes of adrenal crisis, or demographic factors associated with it, may be under-reported by this study. Anecdotally, delays in self-injection or seeking medical assistance during episodes of vomiting – from any cause – appear to be a recurring feature of deaths among diagnosed adrenal patients while “simple” steroid decompensation from running out of replacement therapy has also been recorded as a cause of adrenal crisis triggering organ failure [16, 19].

## Conclusions

This study found a significant increase in the proportion of adrenal patients whose episodes of near-crisis symptomatic deterioration were treated in a timely manner in a pre-hospital setting, both over the 5 year interval of the 2 patient surveys it reports and when compared with earlier patient surveys reported elsewhere. By the time of the 2017–18 survey, around 70% of all episodes where UK adrenal patients reported they had needed injected steroids were initially treated in a pre-hospital setting.

This represents a promising advance in patient safety and appears to be underpinned by several simultaneous initiatives: greater efforts across endocrine units to ensure their adrenal patients receive instruction in self-injection, greater availability of online education materials about injection method and efforts by ambulance trusts to ensure their crews are trained in responses to the steroid-dependent patient and equipped with injectable hydrocortisone. This study suggests that systematic programmes to ensure all adrenal patients are well educated in self-treatment for prevention of adrenal crisis, and are equipped with both generous supplies of oral glucocorticoid medication and injection materials for home use in the event of vomiting, can make a measurable impact on patient safety.

However, this study also indicates that around one-third of adrenal patients experience sub-optimal treatment at emergency medical departments, with a time to treat of more than an hour that could see their condition deteriorate into potentially irreversible circulatory complications. In mitigation, despite increasing resource constraints across the NHS, this proportion has remained broadly constant over the past five years.

For even the largest hospitals, individual medical, paramedical and nursing staff have a low probability of encountering an adrenal crisis, and are likely to be unfamiliar with the specific treatment response it demands. This increases the responsibilities of their employing trusts to ensure that detailed and specific protocols are accessible to all staff, and that adequate training is provided in how to respond to this infrequently encountered – but acutely life-threatening – scenario.

## Data Availability

Excel file logs of all survey responses are available on reasonable request to the corresponding author.

## Abbreviations

AMEND: Association for Multiple Endocrine Neoplasia Disorders
CAH: Congenital adrenal hyperplasia
IM: Intramuscular
IV: Intravenous
NHS: National Health Service
ONS: Office for National Statistics
PAI: Primary adrenal insufficiency
PCA: Prescription Cost Analysis
SAI: Secondary adrenal insufficiency
SC: Subcutaneous
